# Bioinformatics and experimental unveiling of TIMP1 as a novel therapeutic target in colorectal cancer ferroptosis

**DOI:** 10.3389/fonc.2025.1593107

**Published:** 2025-07-04

**Authors:** Chao Tang, Zhenhan Li, Tianyu Song, Linming Lu, Bofeng Chen, Tao Zhang, Yi Zhang, Jie Yang, Jianle Lao, Hao Chen

**Affiliations:** ^1^ School of Medicine, Nanjing University of Chinese Medicine, Nanjing, China; ^2^ School of Clinical Medicine, Wannan Medical College, Wuhu, China; ^3^ The Second Affiliated Hospital, Guangzhou Medical University, Guangzhou, China; ^4^ Department of Pathology, Wannan Medical College, Wuhu, China; ^5^ School of Public Health, Wannan Medical College, Wuhu, China; ^6^ School of Nursing, Wannan Medical College, Wuhu, China; ^7^ Department of Cardiothoracic Surgery, Nanjing Drum Tower Hospital, Affiliated Hospital of Medical School, Nanjing University, Nanjing, China; ^8^ Key Laboratory of Tumor Molecular Pathology of Baise, Department of Cardiothoracic Surgery, Affiliated Hospital of Youjiang Medical University for Nationalities, Baise, China; ^9^ Postdoctoral Research Station of Clinical Medicine, Jinan University, Guangzhou, China; ^10^ Guangxi Technology Innovation Cooperation Base of Prevention and Control Pathogenic Microbes With Drug Resistance, Youjiang Medical University for Nationalities, Baise, China

**Keywords:** colorectal cancer, ferroptosis, TIMP1, tumor malignancy, therapeutic target

## Abstract

**Introduction:**

Colorectal cancer (CRC) remains a globally prevalent and lethal malignancy, with ferroptosis emerging as a novel cell death mechanism. This study aimed to elucidate the role of key genes in ferroptosis regulation and their impact on CRC malignancy.

**Methods:**

Using bioinformatics and experimental methods, we identified TIMP1 as an oncogene that may promote CRC progression via ferroptosis, a pathway implicated in diverse diseases. TIMP1 expression was analyzed in TCGA CRC datasets and validated using the UALCAN database.

**Results:**

TIMP1 was demonstrated as a critical ferroptosis-related gene in CRC, with elevated expression correlating with advanced pathological staging. Immunohistochemistry demonstrated significantly higher TIMP1 levels in CRC tissues compared to healthy controls. Functional assays revealed that TIMP1 knockdown enhanced ferroptosis sensitivity, suppressed CRC cell proliferation and migration, and reduced expression of ferroptosis regulators GPX4 and SLC7A11.

**Conclusion:**

These findings indicate that TIMP1 drives CRC malignancy through ferroptosis modulation, positioning TIMP1 as a potential therapeutic target and offering novel insights for CRC-targeted therapies.

## Introduction

Colorectal cancer (CRC) remains one of the most prevalent and deadly malignancies worldwide, emphasizing the need for innovative approaches to improve diagnosis, prognosis, and treatment (1). Recent research has unveiled a novel cell death mechanism, ferroptosis, as a potential therapeutic target in cancer (2–5). Ferroptosis, characterized by iron-dependent lipid peroxidation and distinct morphological features, differs from traditional apoptosis and necrosis pathways, adding to the complexity of cancer biology (6–8). Studies have explored ferroptosis-related genes in CRC, but the comprehensive understanding of this phenomenon and its clinical relevance remains incomplete.

TIMP1 (Tissue Inhibitor of Metalloproteinases 1) has been studied in the proliferation, drug resistance, and metastasis of malignant tumors (9–11). Tian et al. found that TIMP1 can lead to the proliferation and gemcitabine resistance of pancreatic cancer cells through the mediated PI3K/AKT/mTOR axis (12). Several studies have shown that TIMP1 can promote the invasion and metastasis of pancreatic cancer cells to the liver and perineural areas (13, 14). Current research on the relationship between TIMP1 and ferroptosis primarily focuses on areas such as liver fibrosis and ulcerative colitis. For instance, Li et al. found that Schisandrin B inhibits liver fibrosis by inducing ferroptosis in lymphocyte antigen 6 complex locus C low (Ly6C^lo^) macrophages and hepatic stellate cells, a process that involves the downregulation of TIMP1 (15). Lai et al. have conducted research revealing that Vitamin A-functionalized SF-siHSP47@VFPL-NPs efficiently target activated hepatic stellate cells (aHSCs), resulting in the reduction of TIMP-1 and collagen I levels (16). Multiple studies, utilizing bioinformatics, machine learning, clinical database analysis, and experimental validation, have found that TIMP1 is involved in regulating the ferroptosis process in ulcerative colitis and can serve as a biological indicator for its diagnosis and prediction (17–19). Ulcerative colitis is recognized as a precancerous condition for colorectal cancer (CRC), yet the specific mechanism by which TIMP1 mediates the malignant progression of CRC through modulation of ferroptosis remains unclear (20).

To address this problem, we employed a multifaceted approach. Initially, we conducted differential expression analysis and constructed a ferroptosis-related risk model using The Cancer Genome Atlas (TCGA) data (21). Subsequently, weighted gene co-expression network analysis (WGCNA) was employed to identify key modules associated with ferroptosis, revealing potential therapeutic targets (22). This study uniquely integrates bioinformatics and experimental approaches to unravel the role of TIMP1 in ferroptosis and its impact on CRC malignancy, offering new insights into targeted therapies

## Materials and methods

### Obtaining ferroptosis genes differentially expressed in colorectal cancer based on FerrDb database

We systematically analyzed ferroptosis-related genes from the FerrDb database, selecting genes with stringent criteria (|logFC| > 1, adjusted P < 0.05) to ensure their close association with ferroptosis. To elucidate biological pathways influenced by these differentially expressed genes, functional enrichment analysis was performed using the clusterProfiler R package (v3.14.3), which supports Gene Ontology (GO), Kyoto Encyclopedia of Genes and Genomes (KEGG), and statistical visualization. Gene identifier conversions (e.g., Entrez ID to gene symbol) were facilitated by the org.Hs.eg.db annotation database (v3.10.0). To infer regulatory directionality of enriched terms, we integrated logFC values and computed z-scores using the formula: *Z*-score = (Up-regulated genes−Down-regulated genes)/√gene counts. This metric preliminarily distinguishes positive or negative regulation of pathways.

### Constructing a consensus clustering and ferroptosis prognostic model based on Lasso regression

Lasso regression, an extension of linear regression, mitigates model overfitting and enhances generalization by introducing penalty terms. In our study, Lasso regression was applied to the differentially expressed ferroptosis genes. Subsequently, the gene expression levels and regression coefficients from this analysis were utilized to construct a risk score. For the Consensus Clustering, we employed the ConsensusClusterPlus R package (23). We conducted agglomerative pam clustering using 1-Pearson correlation distances, resampling 80% of the samples for 10 repetitions. The optimal number of clusters was determined through the empirical cumulative distribution function plot. This approach allowed us to develop a robust prognostic model for ferroptosis based on Lasso regression, which was further validated through Consensus Clustering analysis.

### Construction of co-expression networks and gene set variation analysis based on colorectal cancer subtypes

To explore the key genes contributing to the subtypes of CRC, a Weighted Gene Co-expression Network Analysis (WGCNA) was constructed based on subtype grouping. Firstly, using gene expression profiles, the Median Absolute Deviation (MAD) for each gene was calculated and the bottom 50% of genes were excluded with the lowest MAD values. Next, WGCNA was utilized to construct a scale-free co-expression network. After selecting a power of 12, we transformed the adjacency matrix into a topological overlap matrix (TOM) to measure the network connectivity of each gene. The dissimilarity (1-TOM) was then calculated. To cluster genes with similar expression profiles into modules, we performed average linkage hierarchical clustering based on the TOM-based dissimilarity measure. The minimum module size was set to 30 for the genes dendrogram. The sensitivity was set to 3. Furthermore, we calculated the dissimilarity of module eigengenes, determined a cut-off for module dendrogram, and merged some modules. Additionally, we merged modules with a dissimilarity less than 0.25. For Gene Set Variation Analysis (GSVA), we utilized the GSVA R package (version 1.40.1) to compute the enrichment scores for each sample within predefined gene sets. We established gene rank scores initially using gene expression profiles (24). We predefined gene sets to assess the enrichment scores, with a minimum gene set size of 5 and a maximum gene set size of 5000. These calculations resulted in enrichment scores for each sample in each gene set. Finally, we employed the Hallmark database as the reference dataset.

### Exploring clinical characteristics of TIMP1 based on colorectal cancer data in TCGA

We analyzed the expression differences of TIMP1 in both non-paired and paired samples, with data sourced from UCSC XENA. We also conducted a validation using UALCAN. Survival curve plotting was done using transformed data from TCGA, and R packages included survminer (version 0.4.9) and survival (version 3.2-10). Prognosis types included Overall Survival, Disease-Specific Survival, and Progress-Free Interval. We confirmed the transcriptional and protein levels of TIMP1 in both HPA and UALCAN databases.

To systematically characterize the tumor immune microenvironment, we performed immune infiltration analysis using 24 functionally annotated immune cell types, encompassing both innate and adaptive immunity. Immune infiltration scores were quantified via the single-sample Gene Set Enrichment Analysis (ssGSEA) algorithm implemented in the GSVA R package (v1.40.1). This non-parametric method calculates sample-specific enrichment scores by rank-normalizing gene expression matrices and evaluating the cumulative distribution shifts of predefined immune gene sets. Immune cell types included aDC (activated DC), B cells, CD8 T cells, Cytotoxic cells, DC, Eosinophils, iDC (immature DC), Macrophages, Mast cells, Neutrophils, NK CD56 bright cells, NK CD56dim cells, NK cells, pDC (Plasmacytoid DC), T cells, T helper cells, Tcm (T central memory), Tem (T effector memory), Tfh (T follicular helper), Tgd (T gamma delta), Th1 cells, Th17 cells, Th2 cells, and Treg.

### Cell lines and cell culture

The HCT-116 and SW480 CRC cell lines were obtained from the American Type Culture Collection (ATCC). Both cell lines were maintained according to ATCC’s recommended protocols and were regularly tested for contamination and viability to ensure their authenticity and suitability for research purposes. The human CRC cell lines HCT-116 and SW480 were cultured in RPMI-1640 medium (Biological Industries, Israel). The culture medium was supplemented with 10% fetal bovine serum (Gibco, USA), 1% penicillin and streptomycin (Beyotime Biotechnology, China). CRC cells were cultured at 37°C in a 5% CO_2_ incubator.

### Immunohistochemistry

IHC of paraffin-embedded tissue sections from both human normal and CRC specimens were executed according to standard protocols (25, 26), involving deparaffinization, rehydration, and antigen retrieval. Blocking with hydrogen peroxide and goat serum preceded overnight incubation at 4°C with the TIMP1 primary antibody (Proteintech, China; Cat. no. 16644-1-AP) to ensure robust binding. The next day, a secondary antibody was applied for 45 min at 37°C, followed by a 30-min incubation with SABC Reagent solution at the same temperature. Visualization of the antigen-antibody complex was achieved through incubation with 3, 3’-diaminobenzidine (DAB), and light counterstaining with hematoxylin provided contrast. Images were then captured under an inverted fluorescence microscope, allowing for detailed examination of the immunohistochemical staining patterns in both normal and tumor tissues.

### Transient siRNA transfection

Commercially synthesized siRNA targeting TIMP1 (siTIMP1) (sense: 5′→3′ UCAUAACGCUGGUAUAAGGUG; antisense: 5′→3′ CCUUAUACCAGCGUUAUGAGA) and scrambled negative control siRNA (siControl) were procured from Shanghai GenePharma Co., Ltd. (Shanghai, China). For transfection, HCT-116 and SW480 cells were seeded to reach ~70% confluency prior to treatment. siRNA duplexes (100 nM final concentration) were delivered into cells using Lipofectamine Transfection Reagent (cat. no. 40802ES03, Yeasen Biotechnology Co., Ltd., Shanghai, China). Following a 6-hour incubation period, the transfection medium was replaced with fresh complete culture medium. Cells were used for the subsequent experiments.

### Lentiviral transduction

Plasmids containing human TIMP1 cDNA and TIMP1 shRNA cassettes were commercially obtained from Hunan Fenghui Biotechnology Co., Ltd. (Changsha, China). The TIMP1 coding sequence was subcloned into a pCDH vector (Hunan Fenghui Biotechnology Co., Ltd.), while TIMP1-targeting shRNA was inserted into a pLKO vector (Hunan Fenghui Biotechnology Co., Ltd.) under the control of a tetracycline-inducible promoter. The packaging system (comprising VSVG, PLP1, and PLP2 plasmids) and expression vectors were co-transfected into HEK293T cells using Lipofectamine Transfection Reagent for lentivirus production. Control refers to HCT-116 cells transduced by empty lentiviral plasmids. Viral supernatant was harvested 48 h post-transfection and cryopreserved at −80°C. Transduced HCT-116 cells were subsequently selected with tetracycline to ensure high transduction efficiency.

### Colony formation assay

For the colony formation assay, HCT-116 or SW480 CRC cell lines were meticulously plated into 6-well plates at a density of 10^3^ CRC cells per well. This ensured that the cells had adequate space to proliferate and form colonies over the course of the experiment. Upon completion of the experimental period, the colonies were meticulously washed with PBS buffer to remove any cellular debris or media components that could interfere with subsequent staining steps. Next, the colonies were fixed using 4% paraformaldehyde for a duration of 20 min. This step was crucial for preserving the cellular structure and morphology, allowing for accurate visualization and enumeration of the colonies. Following fixation, the colonies were stained with crystal violet (Beyotime Biotechnology, China, Cat. no. C0121) for 20 min, and finally washed twice with PBS buffer. Crystal violet is a commonly used dye in colony formation assays due to its ability to stain cellular components, such as DNA and proteins, providing a clear and visible contrast against the background. CRC cells colonies stained with crystal violet were finally observed and counted under a microscope

### Apoptosis analysis

Approximately 10^6^ CRC cells were meticulously washed three consecutive times with PBS buffer to eliminate any residual culture media or reagents that might interfere with the subsequent staining and analysis steps. Following the washing procedure, the CRC cells were gently resuspended. Subsequently, the resuspended CRC cells were incubated with a cocktail of propidium iodide (PI) and Annexin V dyes for a duration of 20 min in a controlled environment, allowing sufficient time for the dyes to bind to their respective targets. PI, a fluorescent dye that stains the DNA of dead or dying cells, permeates the membranes of apoptotic and necrotic cells, while Annexin V, a marker of early apoptosis, binds specifically to phosphatidylserine residues exposed on the outer leaflet of the plasma membrane of apoptotic cells. After the incubation period, the stained CRC cells were carefully aspirated and introduced into a flow cytometer (manufactured by BD Biosciences, USA) for comprehensive analysis.

### Transwell assay

HCT-116 or SW480 CRC cells (10^5^ cells per transwell chamber) were seeded into 8 μm pore upper chambers with serum free medium, and then cultured with complete medium in the lower chamber. After 24 h, cells were washed with PBS, fixed with 4% paraformaldehyde for 20 min, and subsequently stained with crystal violet for 20 min. Finally, the crystal violet dye and cells in the upper chamber were scraped with a cotton swab, and the migration of the cells in the lower chamber was observed with a microscope.

### Western blot

The cells were harvested and meticulously washed with PBS buffer to remove any contaminants or media residues. Subsequently, RIPA lysate was employed to effectively extract the total cellular proteins. Protein concentrations were determined using a BCA Protein Assay Kit (Thermo Fisher Scientific, Cat. no.23227). Equal amounts of protein lysates were separated by 10% SDS-PAGE and electrophoretically transferred to PVDF membranes (Millipore, Cat. no. ISEQ00010). After blocking with 5% skimmed milk for 1 h at room temperature, membranes were incubated overnight at 4°C with the following primary antibodies: TIMP1 (Proteintech, China; Cat. no.16644-1-AP), GPX4 (Proteintech, China; Cat. no. 30388-1-AP), SLC7A11 (Proteintech, China; Cat. no.26864-1-AP), and β-actin (Proteintech, China; Cat. no.66009-1-Ig). Following three 10-min washes with TBST (Tris-buffered saline containing 0.1% Tween-20), membranes were incubated with appropriate HRP-conjugated secondary antibodies for 90 min at room temperature. Protein bands were visualized using enhanced chemiluminescence (ECL) reagent (Thermo Fisher Scientific, Cat. no.34580) and imaged with a chemiluminescence detection system.

The methodology for determining the density of protein expression is as follows: The WB bands were imported into ImageJ software for grayscale value analysis, and the resultant data were subsequently statistically analyzed using GraphPad Prism 8 software. Given that the WB experiments were performed in triplicate, an initial step involved calculating the mean grayscale value for the three replicates in the first group, designated as the control group. Following this, the grayscale values of all other groups were normalized by dividing them by this mean control value, ultimately yielding a relative protein expression quantity for each group. This approach allowed for a precise and comparable assessment of protein expression levels across different experimental conditions.

### Subcutaneous CRC xenograft model

The 6-week-old nude mice were purchased and raised in the Laboratory Animal Center of Wannan Medical College. The animals were kept in constant environmental conditions, with 12-hour light and dark cycles and free access to food and water. In this experiment, none of the mice showed infections, wounds, or significant weight loss. Control and stable TIMP1 knockdown (TIMP1-KD) HCT-116 cells (10^6^ cells) were subcutaneously injected into the 6-week-old BALB/c nude mice. The tumor growth was monitored at 3-day intervals, with tumor volume calculated using the formula: length × (width)^2/2. In the colorectal cancer xenograft tumor model, mice were anesthetized with 1.5% isoflurane by inhalation when their tumors reached a diameter of 15 mm (National Cancer Institute, Guidelines for Tumor Burden in Rodent Models). For more details, refer to the source (https://www.cancer.gov/). All animal experiments in this study were conducted according to the ARRIVE guidelines (https://arriveguidelines.org/arrive-guidelines). All procedures involving animals were also approved by the Animal Welfare and Ethical Committee of Wannan Medical College.

### Statistical analysis

GraphPad Prism Software (version 8.0.1) was used for statistical analysis. All comparisons between 2 groups were performed using two tailed unpaired Student’s t test. Three or more groups were compared using one-way ANOVA analysis. *P*<0.05 was statistically significant.

## Results

### Landscape of differentially expressed ferroptosis gene in TCGA-COAD

**Figure 1:**
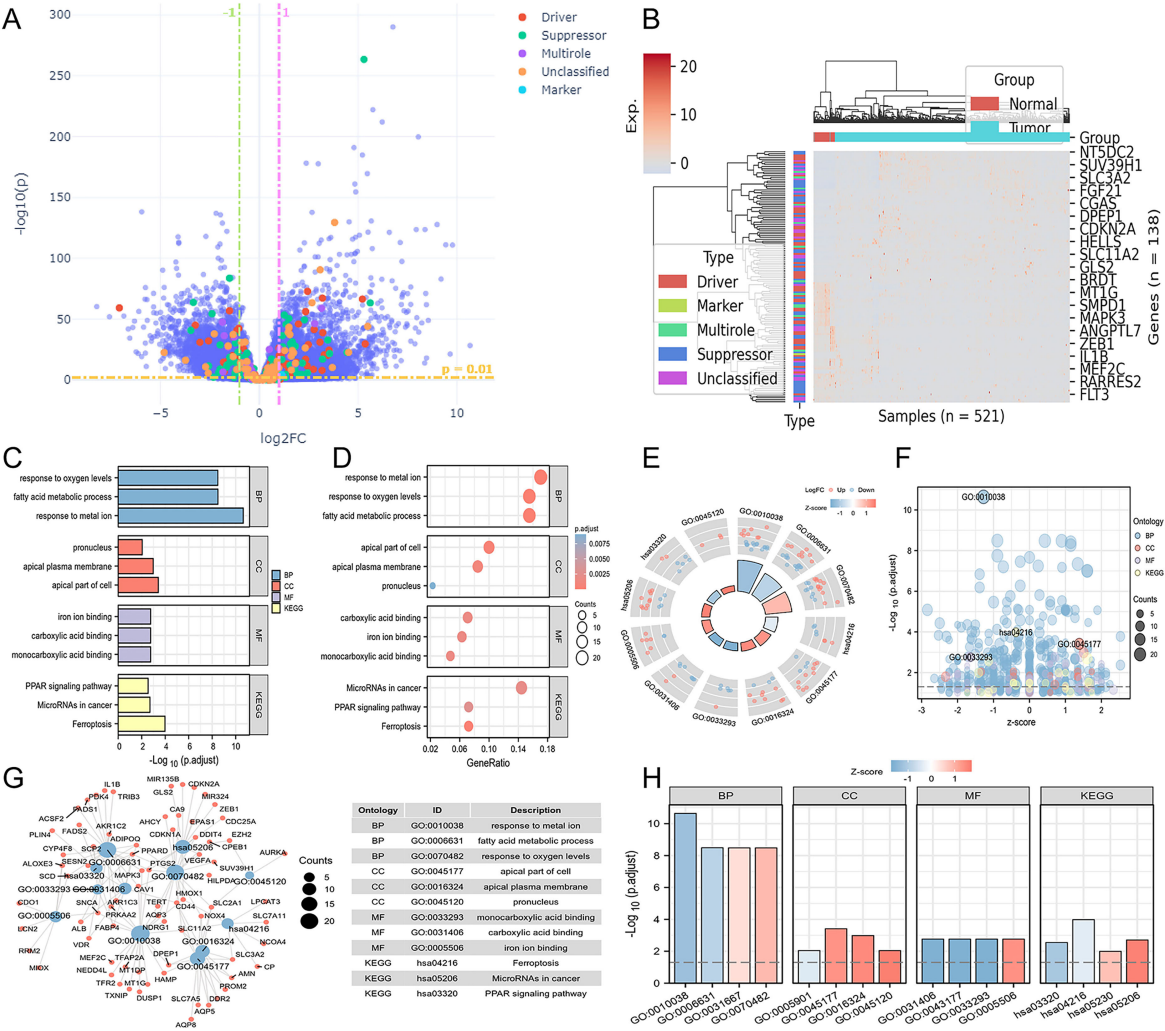
Differential landscape and enrichment analysis of TCGA-COAD. **(A)** Volcano plot illustrating differential changes in iron-related death-associated genes. **(B)** Heatmap depicting the landscape of differential gene expression profiles. **(C)** Bar chart presenting GO/KEGG enrichment results. **(D)** Bubble chart displaying GO/KEGG enrichment results. **(E)** Circular plot combining logFC to compute z-scores for entries. **(F)** Bubble chart exhibiting z-scores for each entry. **(G)** Visual network representation between entries and differential genes associated with iron death. **(H)** Bar chart displaying z-scores for the most significant entries.

We obtained manually curated ferroptosis-related genes from the FerrDb database, classifying them into five categories based on their distinct biological functions: Driver, Suppressor, Multirole, Unclassified, and Maker. Hierarchical clustering was performed on differentially expressed regulators of ferroptosis genes, and expression values were z-scored. After conducting differential analysis on CRC samples from TCGA, the specific information about the patient samples was shown in **Supplementary Table 1**, and a total of 139 differentially expressed genes that met our threshold criteria were identified (as shown in **Supplementary Table 2**). We visualized the details of these differentially expressed genes using heatmaps and volcano plots (**Figures 1A, B**). Subsequently, we performed GO/KEGG enrichment analysis on the differentially expressed ferroptosis genes. This analysis revealed a significant enrichment of biological pathways related to ferroptosis in CRC. In particular, in the KEGG analysis, we observed significant enrichment in several key biological pathways, including “ferroptosis,” “microRNAs in cancer,” and the “PPAR signaling pathway” (**Figures 1C, D**). Additionally, by calculating the z-scores based on the expression characteristics of the differentially expressed genes, we found the further indications that ferroptosis and PPAR signaling pathway may be negatively regulated in colorectal cancer, while “MicroRNAs in cancer” may be significantly activated (**Figures 1E–H**).

### Constructing risk scores and disease subtypes by Lasso regression

**Figure 2:**
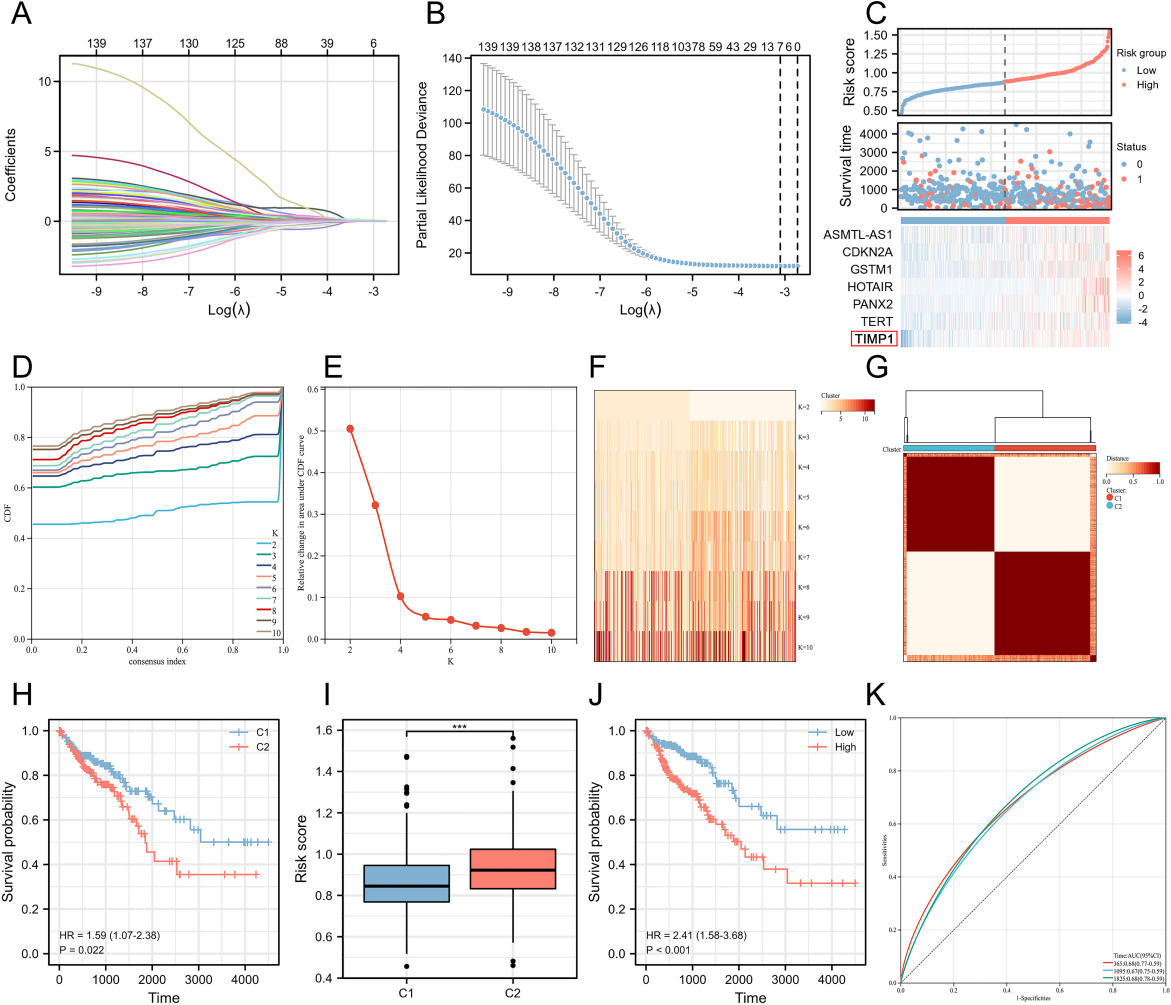
Construction of colorectal cancer disease subtypes based on ferroptosis-related key genes. **(A)** Lasso variable trajectory plot. **(B)** Seven selected variables via Lasso. **(C)** Risk factor plot combining risk score. **(D)** Cumulative distribution curve. **(E)** Area under the distribution curve. **(F)** Sample clustering consistency under different cluster numbers. **(G)** Heatmap of sample clustering consistency when the cluster number is set to 2. **(H)** Survival curve based on disease subtypes. **(I)** Comparison of risk scores in different disease subtypes. **(J)** Survival curve stratified by risk score. **(K)** ROC curve illustrating the stability of the risk score in predicting patient prognosis outcomes at different time points. ***P<0.001.

Lasso regression analysis was performed on the 139 differentially expressed genes related to ferroptosis, utilizing the available clinical data from TCGA. This step helped us eliminate redundant variables and select a few key genes that have the most significant impact on the ferroptosis phenotype and are also associated with patient prognosis. Ultimately, we identified the crucial genes related to ferroptosis (**Figures 2A, B**). After constructing a risk score based on the expression levels and regression coefficients of these ferroptosis-related genes, we observed the expression trends of these genes roughly corresponded to the high and low risk score groups. As the gene expression levels increased, the risk score gradually increased as well (**Figure 2C**). Based on the ferroptosis-related genes enriched from the results, further univariate and multivariate Cox regression analyses were conducted to assess the impact of individual factors (univariate) and their combined effects (multivariate) on survival and recurrence in CRC patients. These analyses yielded hazard ratios (HRs) and corresponding statistical P-values, where an HR > 1 with a P < 0.05 indicates a significant increase in CRC risk. The interpretation of HRs is crucial for understanding the prognostic implications and guiding treatment strategies associated with various factors. Among the ferroptosis-related genes screened, only TIMP1 and TERT met the criteria of HR > 1 and P < 0.05, preliminary suggesting a potential association between these ferroptosis-related genes, TIMP1 and TERT, and the malignant progression of CRC (**Table 1**). We next conducted a comprehensive analysis and evaluation of both TIMP1 and TERT, two ferroptosis-related genes. Leveraging clinical data from CRC patients, we found that in both GSE44861 and GSE44076 datasets, TIMP1 mRNA was significantly overexpressed in tumor tissues compared to healthy normal tissues, whereas no significant difference was observed in TERT expression between normal and CRC tumor groups (**Supplementary Figure S1A-D**). Furthermore, survival analysis of the GSE29623 CRC database revealed that high TIMP1 expression was closely associated with poor survival in CRC patients, whereas TERT expression was not significantly correlated with CRC patient survival (**Supplementary Figure S1E, F**). These results collectively indicate that TIMP1 is closely related to poor outcomes in CRC patients, whereas TERT does not exhibit a significant correlation with poor outcomes in CRC. Therefore, we selected TIMP1 for further investigation in this study.

**Table 1:** COX regression of univariate analysis and multivariate analysis.

Characteristics	Total (N)	Univariate analysis		Multivariate analysis	
		Hazard ratio (95% CI)	*P* value	Hazard ratio (95% CI)	*P* value
ASMTL-AS1	462	1.253 (1.037-1.514)	0.019	1.186 (0.959-1.465)	0.115
CDKN2A	462	1.223 (1.071-1.397)	0.003	1.103 (0.949-1.282)	0.202
GSTM1	462	1.167 (1.047-1.302)	0.005	0.997 (0.817-1.215)	0.973
HOTAIR	462	1.338 (1.120-1.599)	0.001	1.231 (1.018-1.488)	0.032
PANX2	462	1.459 (1.162-1.832)	0.001	1.221 (0.922-1.616)	0.164
TERT	462	1.371 (1.091-1.723)	0.007	1.332 (1.019-1.740)	0.036
TIMP1	462	1.399 (1.121-1.745)	0.003	1.366 (1.059-1.761)	0.016
lasso.risk.score	462	25.751 (8.834-75.068)	<0.001		
Group	462				
C1	244	Reference			
C2	218	1.594 (1.069-2.378)	0.022	1.643 (0.819-3.294)	0.162

Next, we used these key genes related to ferroptosis to create a Consensus Cluster, which allowed us to define disease subtypes based on the expression characteristics of ferroptosis-related genes. We found that the number of clusters was set to 2, resulting in the best sample clustering (**Figures 2D, E**). Consequently, two distinct disease subtypes were identified (**Figures 2F, G**). We grouped the samples based on the previously identified disease subtypes and organized the corresponding clinical data to generate the survival curves. Surprisingly, we discovered that the patients classified in C2 subtype were with significantly the worse prognosis outcomes compared to those in C1 subtype (**Figure 2H**). Additionally, we noted that the risk score was higher in C2 subtype, aligning with our earlier analysis. Therefore, we separately stratified the risk score into high and low groups, revealing that the high-risk score group had significantly worse prognosis outcomes (**Figures 2I, J**). To evaluate the accuracy and robustness of our ferroptosis prognosis model, we assessed the ability of the ferroptosis-related risk score to predict the prognosis of CRC patients by plotting ROC curves. The results showed that our model maintained a stable predictive performance at different time points, including one year, three years, and five years (**Figure 2K**).

### Unveiling key biological pathways underlying induced disease subtype differences through joint WGCNA analysis

**Figure 3:**
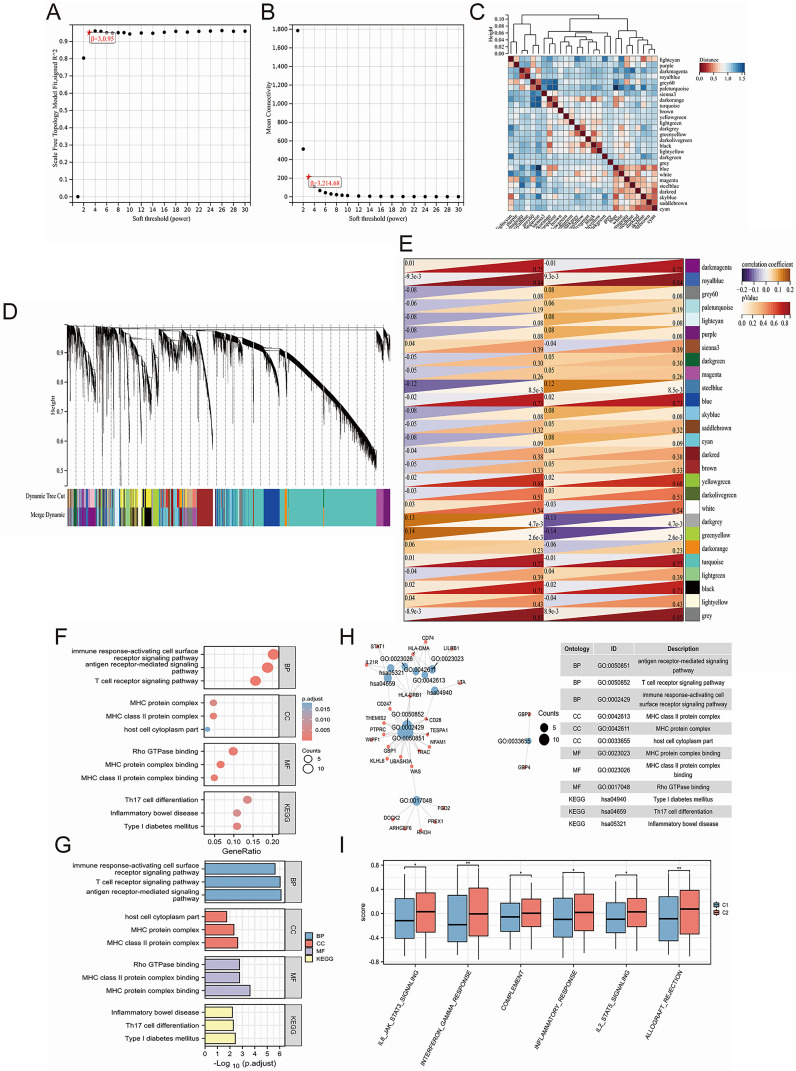
Uncovering key biological pathways underlying differences in induced disease subtypes through joint WGCNA analysis. **(A, B)** Determination of the optimal soft threshold for constructing the co-expression network based on average connectivity and scale independence, with the optimal threshold identified as 3. **(C)** Identification of 27 distinct gene modules using WGCNA. **(D)** Clustering of module eigengenes and genes to illustrate the relationships between different modules and genes. **(E)** Correlation analysis between different modules and disease subtypes, highlighting a significant negative correlation between the steel blue module and the C2 subtype, while the dark gray and green yellow modules show a significant positive correlation with the C2 subtype. **(F-H)** GO/KEGG enrichment analysis of 79 hub genes identified from clinically significant modules, showing enrichment in immune regulation pathways, including T cell and antibody-related biological processes (BP), MHC-related cellular components (CC), and MHC-related molecular functions (MF). **(I)** GSVA analysis revealing significant upregulation of biological pathways in the C2 subtype. *P<0.05, **P<0.01.

WGCNA is able to help identify the potential key genes. Based on the average connectivity and scale independence, the optimal soft threshold for constructing the co-expression network was 3 (**Figures 3A, B**). Subsequently, we obtained 27 different gene modules (**Figure 3C**). We performed module eigengene clustering and gene clustering to illustrate the relationships between modules and genes (**Figure 3D**). Furthermore, we calculated the correlation between different modules and disease subtypes. We found a significant negative correlation between the steel blue module and C2 subtype, while the dark gray and green yellow modules showed a significant positive correlation with C2 subtype (**Figure 3E**).

We calculated the correlation between module eigengenes and gene expression to obtain Module Membership (MM). Based on the cut-off criteria, 79 genes with high connectivity in the clinically significant module as hub genes were identified (as shown in **Supplementary Table 3**). GO/KEGG enrichment analysis on these 79 hub genes was conducted and we found that these genes are related to immune regulation. In BP, we enriched pathways related to T cells and antibodies, such as “antigen receptor-mediated signaling pathway,” “T cell receptor signaling pathway,” and “immune response-activating cell surface receptor signaling pathway.” In CC, we enriched pathways related to the MHC, such as “MHC class II protein complex,” “MHC protein complex,” and “host cell cytoplasm part.” Similarly, in MF, we enriched pathways related to MHC, including “MHC protein complex binding,” “MHC class II protein complex binding,” and “Rho GTPase binding.” Finally, in KEGG pathways, we enriched pathways such as “Th17 cell differentiation,” and “Inflammatory bowel disease” (**Figures 3F–H**). Moreover, through GSVA analysis, we found that some of these biological pathways were significantly upregulated in the C2 subtype, including “IL6-JAK-STAT3 signaling,” “interferon gamma response,” “complement,” “inflammatory response,” “IL2-STAT5 signaling,” and “allograft rejection” (**Figure 3I**).

### Exploring the distribution characteristics of risk scores in different clinical subgroups

**Figure 4:**
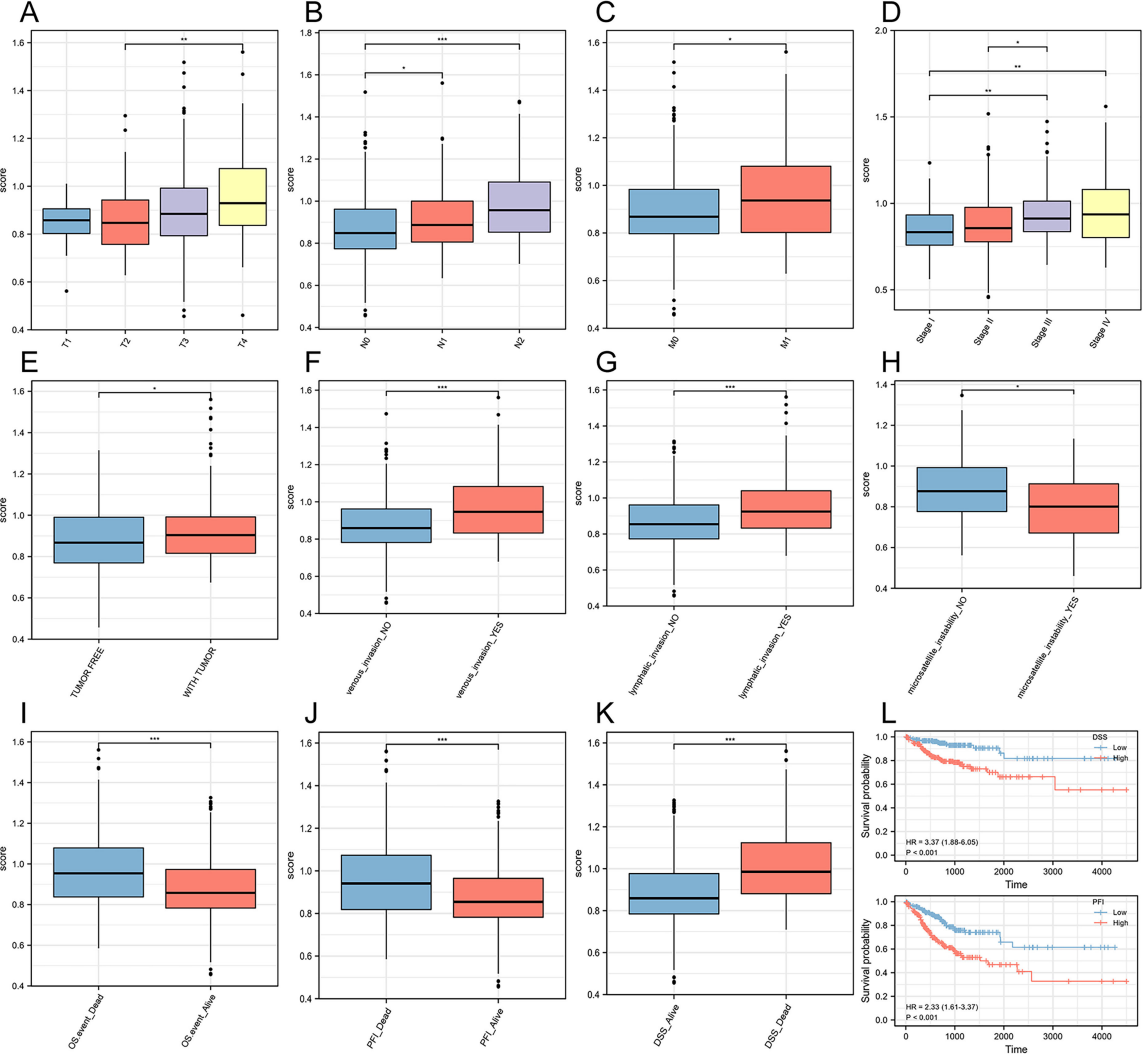
Construction of a risk score prognostic model based on ferroptosis-related genes. **(A)** T stage. **(B)** N stage. **(C)** M stage. **(D)** Pathological stage. **(E)** Person neoplasm cancer status. **(F)** Venous invasion. **(G)** Lymphatic invasion. **(H)** Microsatellite instability. **(I)** Overall Survival (OS). **(J)** Progression-Free Interval (PFI). **(K)** Disease-Specific Survival (DSS). **(L)** Survival curves for two prognosis types. *P<0.05, **P<0.01, ***P<0.001.

To explore the prognostic value of the risk score with respect to clinical staging, we grouped and compared the risk score based on clinical information from TCGA. We observed that in the T stage, patients in the T4 stage had significantly higher risk scores compared to those in the T2 stage (**Figure 4A**). In the N stage, N2 stage patients had the highest risk scores, while N1 stage patients had higher scores than N0 stage patients (**Figure 4B**). These findings suggested that the risk score had clinical values in predicting and indicating lymphatic metastasis in CRC. Similarly, M1 stage patients had higher risk scores than M0 stage patients, indicating that the risk score also had predictive and evaluative capabilities for distant tumor metastasis (**Figure 4C**). Furthermore, the risk score showed significant differences among four different pathological stages, with stage IV having the highest scores (**Figure 4D**). Tumor-free status officially means that no cancer is currently detectable in the body. This determination may be based on scans, bloodwork, or other tests, such as a breast or bone marrow biopsy. Our results showed that the risk score in patients with tumors was significantly higher than in tumor-free patients (**Figure 4E**). Once again, we further proved that the risk score had strong predictive and evaluative capabilities for both hematogenous and lymphatic metastasis. When patients experienced hematogenous or lymphatic metastasis, the risk score significantly increased (**Figures 4F, G**). Furthermore, the risk score also had a certain guiding role in the microsatellite instability of colorectal cancer patients (**Figure 4H**). In three different prognosis types, the dead patients were with the higher risk scores than those alive ones (**Figures 4I–K**). Additionally, in the Disease-Specific Survival (DSS) and Progression-Free Interval (PFI) prognosis types, the high-risk score group had significantly worse prognosis outcomes than the low-risk score group (**Figure 4L**).

### Exploring the expression, prognostic features, and immune characteristics of TIMP1

**Figure 5:**
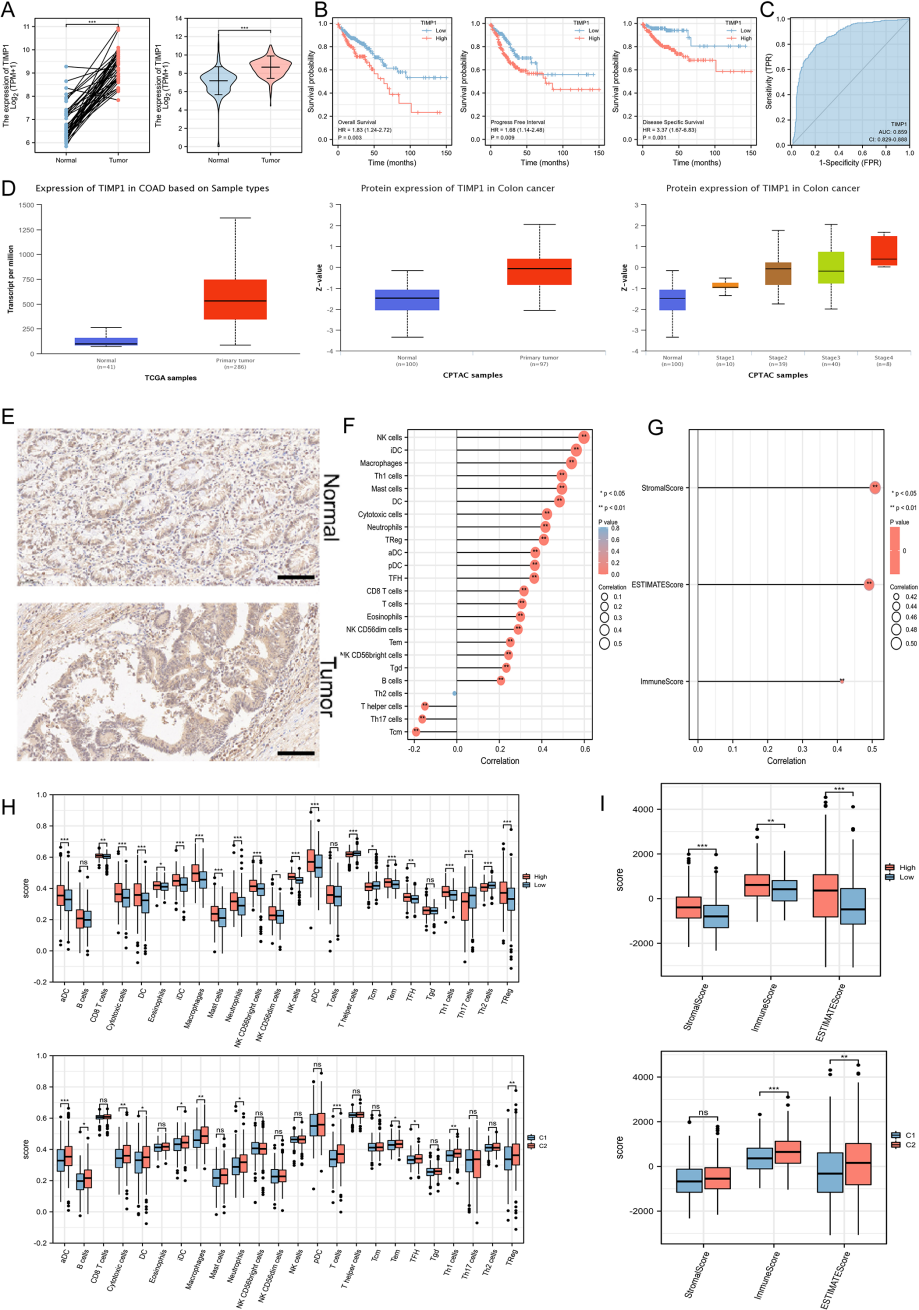
Exploration of the expression characteristics, prognostic features, and immune features of key ferroptosis-related genes. **(A)** The mRNA expression levels of TIMP1 in paired and non-paired samples. **(B)** An increased level of TIMP1 was remarkably associated with poor survival of patients. **(C)** ROC analysis. **(D)** Differential protein levels of TIMP1 in the UALCAN database. **(E)** IHC staining of TIMP1 in normal (n=10) and CRC samples (n=10). Scale bar: 100 μm. **(F)** Correlation of TIMP1 expression levels with the infiltration levels of 24 immune cells. **(G)** ESTIMATE algorithm showing the relationship between TIMP1 expression levels and immune infiltration. **(H)** Exploration of different immune cell infiltration levels based on disease subtypes and ferroptosis risk groupings. **(I)** ESTIMATE algorithm showing differences in immune infiltration based on disease subtypes and ferroptosis risk groupings. *P<0.05, **P<0.01, ***P<0.001. “ns” represented no statistical significance.

Based on the expression matrix in TCGA, we observed that the TIMP1 transcriptional levels in CRC were significantly higher than those in normal tissues (**Figure 5A**). The survival curves also showed that high expression of TIMP1 was closely related to poor survival of CRC patients (**Figure 5B**). Across three different prognosis types, patients with high expression of TIMP1 had much worse prognosis outcomes compared to those with low expression, and ROC analysis confirmed the stability of TIMP1 as a diagnostic molecule for CRC (**Figure 5C**). We further validated the transcriptional differences of TIMP1 in the UALCAN database and found a significant increase in TIMP1 protein levels in CRC patients. Moreover, the protein levels increased with the progression of pathological staging (**Figure 5D**). Immunohistochemistry (IHC) assay demonstrated that TIMP1 was strongly expressed in CRC samples compared with the healthy tissues (**Figure 5E**, **Supplementary Figure S2**). To explore the relationship between TIMP1 and the immune system, the correlation between TIMP1 expression levels and the infiltration levels of different immune cells was calculated. We found that TIMP1 exhibited strong positive correlations with the infiltration levels of most immune cells (**Figure 5F**). Additionally, TIMP1 showed strong positive correlations with stromal score, ESTIMATE score, and ImmuneScore (**Figure 5G**). Subsequently, we investigated the differences in immune infiltration levels among groups stratified by risk score and disease subtypes. We found that the high-risk score and C2 subtype groups had significantly higher immune infiltration scores compared to other groups (**Figures 5H, I**).

### TIMP1 knockdown facilitates the ferroptosis and inhibits the CRC cells proliferation and migration

**Figure 6:**
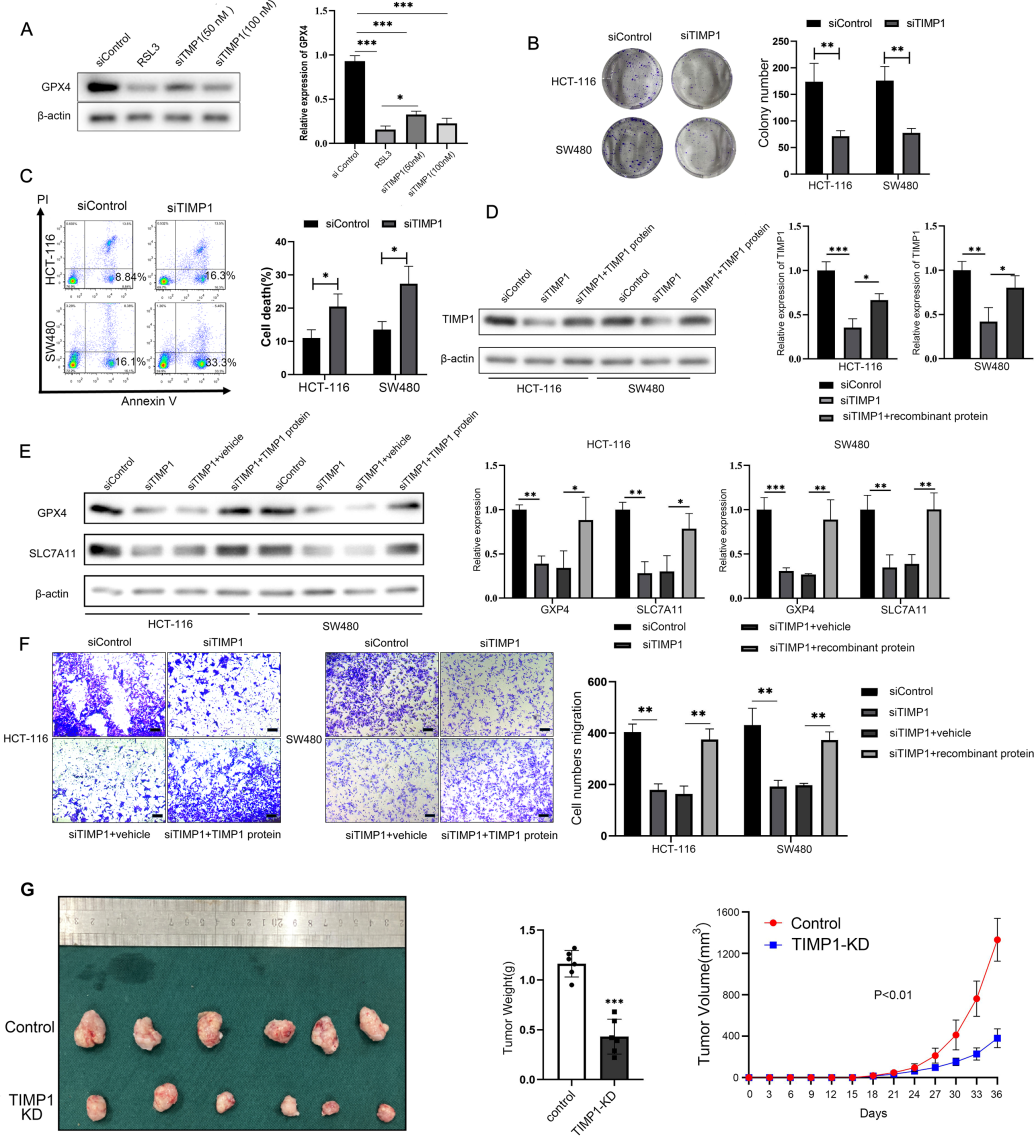
TIMP1 promotes the proliferation and migration of CRC cells by inhibiting ferroptosis. **(A)** WB detected the effect of different transfection concentrations of TIMP1 on the protein expression of GPX4. RSL3 is a specific inhibitor of GPX4, which is used here as a positive control. **(B)** Clonal formation of two CRC cells after transfection of 100 nM TIMP1 siRNA in HCT-116 cells and SW480 cells. **(C)** Flow cytometry was used to detect cell death in two CRC lines after transfection of 100 nM TIMP1 siRNA. **(D)** WB was used to detect the protein expression of TIMP1 after TIMP1 was knocked down or re-adding recombinant TIMP1 protein. **(E)** WB was used to detect the expression of ferroptosis-related proteins GPX4 and SLC7A11 after knockdown of TIMP1 and re-addition of recombinant TIMP1 protein. **(F)** The transwell assay was used to detect the migration ability of CRC cells after knockdown of TIMP1 or re-addition of TIMP1 purified protein. Scale bar: 200 μm. The results of **(A-F)** were from three independent experiments. **(G)** Representative photograph of CRC xenograft tumors following stable TIMP1 knockdown, accompanied by corresponding statistical graphs depicting tumor weight and growth curves (n = 6). *P<0.05, **P<0.01, ***P<0.001.

In order to investigate the regulation of TIMP1 on ferroptosis in CRC cells by specific experiments, we first successfully used siRNA to knock down TIMP1 expression (**Supplementary Figure S3**). Glutathione peroxidase 4 (GPX4) is an antioxidant enzyme necessary to maintain cell REDOX homeostasis, which can inhibit lipid peroxidation and is a key regulator of ferroptosis. In general, its high expression can inhibit ferroptosis process. WB assay showed that knockdown of TIMP1 significantly down-regulated the protein expression of GPX4. In addition, when transfected with 100 nM TIMP1 siRNA into HCT-116 cells, inhibition of GPX4 protein levels was comparable to that of the GPX4-specific inhibitor RSL3 (the positive reference of ferroptosis) (**Figure 6A**). TIMP1 knockdown significantly elevated both MDA and Fe^2+^ levels, consistent with enhanced ferroptosis. Critically, co-treatment with the iron chelator DFO effectively reversed these increases, confirming the ferroptosis-specific effect of TIMP1 in CRC (**Supplementary Figure S4**). Clonal formation assay showed that the cell proliferation rate was found to be significantly decreased in TIMP1 knockdown cells compared with control CRC cells (**Figure 6B**). Flow cytometry assay found that knocking down TIMP1 could significantly induce CRC cell death (**Figure 6C**). The recombinant TIMP1 protein was re-incorporated into the cells that knocked down TIMP1, and WB experiment showed that TIMP1 protein was restored to a higher level (**Figure 6D**). Knockdown of TIMP1 in HCT-116 and SW480 cells significantly induced the decline of ferroptosis-related proteins GPX4 and SLC7A11, while the addition of recombinant TIMP1 protein effectively promoted their recovery (**Figure 6E**). We conducted CCK8 cell proliferation assays, confirming that TIMP1 knockdown could effectively curb the proliferation of CRC cell lines, while purified TIMP1 protein could re-establish the elevated proliferative ability of CRC cells (**Supplementary Figure S5**). Transwell experiment demonstrated that the migration of CRC cells could be effectively inhibited by weakening the expression of TIMP1, while re-addition of recombinant TIMP1 protein significantly reversed the inhibition of cell migration caused by downregulation of TIMP1 (**Figure 6F**). Following TIMP1 knockdown (siTIMP1), we observed a marked increase in the expression of ferroptosis-related proteins GPX4 and SLC7A11, while co-treatment with DFO significantly reversed this upregulation, restoring GPX4 and SLC7A11 levels (**Supplementary Figure S6**). Finally, we successfully established TIMP1-stably knockdown CRC cell lines via lentiviral transduction and further constructed the xenograft model using these knockdown cells to validate the critical role of TIMP1 in the malignant growth of CRC *in vivo* (**Supplementary Figure S7**). The xenograft tumor model also indicated that down-regulated TIMP1 markedly blunted tumor growth (**Figure 6G**). The WB assay also demonstrated a significant downregulation of GPX4 and SLC7A11 expression in TIMP1-KD tumors compared to controls, consistent with enhanced ferroptosis activation following TIMP1 depletion (**Supplementary Figure S8**). Collectively, TIMP1 is able to promote CRC cellular proliferation and migration by regulating ferroptosis process.

## Discussion

Ferroptosis is a novel cell death form distinct from the traditional cell apoptosis and plays a significant role in CRC (27). However, the mechanism regulating ferroptosis in CRC cells remains unclear. In present study, we first collected the differentially expressed genes related to ferroptosis in CRC and conducted GO/KEGG enrichment analysis, which showed that the signaling pathway associated with ferroptosis was significantly inhibited in CRC. In addition, the landscape ferroptosis related gene analysis showed in detail: in the Biological Process (BP) category, the pathways associated with fatty acid metabolism and oxidative stress were identified, such as “response to metal ion”, “fatty acid metabolic process”, and “response to oxygen levels”. In the Cellular Component (CC) category, we found subcellular locations, including “apical part of cell,” “apical plasma membrane,” and “pronucleus.” In the Molecular Function (MF) category, the results suggested unique molecular functions, such as “monocarboxylic acid binding,” “carboxylic acid binding,” and “iron ion binding.” Notably, many of the above processes were related to TIMP1, which suggested that TIMP1 was involved in CRC progression by regulating ferroptosis. Subsequently, we employed Lasso regression analysis to identify seven key ferroptosis-related genes. Based on their gene expression levels and regression coefficients, a risk score was calculated, constructing a colorectal cancer-related ferroptosis risk model that exhibited good stability in predicting patient prognosis.

Furthermore, utilizing the expression profiles of these key ferroptosis-related genes, the consensus clustering was performed to unearth different disease subtypes, ultimately identifying two distinct subtypes with significant differences in prognosis. Using these subtypes, WGCNA was constructed to explore key driver genes between subtypes. Subsequent enrichment analysis revealed that these genes played critical roles in the immune system, such as Th17 cell differentiation. Current research has implicated a close correlation between key ferroptosis-related genes such as TIMP1, CAV1, CD44, HIF1A, and IFNG, and immune infiltration in Crohn’s disease (28). However, studies on the involvement of TIMP1 in regulating immune cells and immune infiltration-mediated CRC are relatively scarce. Intriguingly, our preliminary findings have unveiled a process by which TIMP1 mediates Th17 cell immune infiltration in CRC, suggesting a pivotal role for TIMP1 within the immune microenvironment of CRC. We then applied gene set variation analysis (GSVA) between disease subtypes, uncovering several significantly activated pathways in the C2 subtype, all of which had pro-carcinogenic effects.

The efficiency of the ferroptosis prognosis model was assessed in predicting clinical features of CRC. As TNM and pathological staging advanced, the risk score also significantly increased. Additionally, the prognosis model could predict the status of hematogenous and lymphatic metastasis in colorectal cancer. Through multifactorial COX regression analysis, we identified TIMP1 as a crucial ferroptosis prognosis gene in colorectal cancer. TIMP1 has been proved to be an oncogene in malignant tumors, especially in the proliferation, metastasis, drug resistance, stemness and other processes of digestive tract tumors (14, 29–32). Our results also suggested that TIMP1 promoted the CRC cellular proliferation and metastasis process by regulating the ferroptosis pathway. Furthermore, some studies have found that TIMP1 in serum can be used as a biomarker for the prediction and diagnosis of malignant tumors such as colorectal cancer (33), gastric cancer (34), oropharyngeal cancer (35), etc., which fully indicates that TIMP1 has potential clinical practice value and guiding role in malignant tumors. Our results also further supplement the importance of TIMP1 in predicting the occurrence and development of colorectal cancer.

The main limitation of this study is that the specific molecular mechanism through which TIMP1 inhibits the ferroptosis process of CRC cells has not been effectively verified. Next, we will continue to explore the specific mechanism of TIMP1 regulating ferroptosis in CRC, and confirm the scientificity and reliability of TIMP1 as an effective target for CRC prevention, diagnosis and treatment through multiple ways such as bioinformatics analysis, basic experiment and clinical observation.

While TIMP1 shows promise as a therapeutic target in CRC, its physiological roles in normal tissues warrant careful consideration. TIMP1 is produced by dendritic cells and plays a critical role in enhancing antitumor immune responses by activating immune cells (36). However, systemic inhibition of TIMP1 may disrupt immune surveillance or promote inflammation, as elevated TIMP1 levels correlate with neutrophil-driven inflammatory diseases. In pancreatic cancer, TIMP1 promotes neutrophil extracellular trap formation, driving tumor progression, yet its absence may impair immune defenses (32). Similarly, in liver cancer, TIMP1 paradoxically facilitates metastasis by modulating the hepatic microenvironment, underscoring context-dependent effects (37). These findings suggest that TIMP1-targeted therapies in CRC may require tissue-specific delivery strategies to minimize off-target effects. Unlike its role as a pro-tumorigenic factor in CRC, TIMP1 exhibits dual functionality in pancreatic cancer. While it correlates with poor prognosis in pancreatic ductal adenocarcinoma (PDAC), TIMP1 also promotes neutrophil-mediated immune suppression, complicating therapeutic targeting (32). In liver cancer, TIMP1 paradoxically enhances metastatic niche formation by recruiting neutrophils, despite its canonical role as a metalloproteinase inhibitor (37). Conversely, in CRC, our data demonstrate that TIMP1 directly suppresses ferroptosis and drives malignancy, with minimal prior evidence linking it to neutrophil recruitment. These differences highlight the need for cancer-specific mechanistic studies to guide TIMP1-based interventions. The specificity of TIMP1 as a CRC driver is supported by its correlation with pathological staging and its overexpression in tumor tissues compared to healthy controls (as shown in **Figure 3**). Unlike in ulcerative colitis, where TIMP1 upregulation contributes to matrix remodeling but not directly to malignancy (38), our functional assays confirm that TIMP1 knockdown selectively impairs CRC cell proliferation and ferroptosis resistance. These findings align with studies implicating TIMP1 in CRC metastasis via MMP inhibition (37), further distinguishing its role in CRC from other malignancies.

In conclusion, our study reveals that TIMP1 promotes CRC malignancy by inhibiting ferroptosis, suggesting its potential as a therapeutic target. Through comprehensive bioinformatics and experimental approaches, we demonstrate that TIMP1 is highly expressed in CRC tissues and is associated with poor patient survival. Knockdown of TIMP1 significantly down-regulates ferroptosis-related proteins, inhibits CRC cell proliferation and migration, and promotes CRC cell ferroptosis. These findings underscore the importance of TIMP1 in CRC progression and offer promising avenues for targeted therapies.

## Data Availability

The datasets presented in this study can be found in online repositories. The names of the repository/repositories and accession number(s) can be found below: https://www.ncbi.nlm.nih.gov/geo/, GSE136394.

## Supplementary material

Supplementary Figure 1 Elevated expression of TIMP1, rather than TERT, is closely correlated with adverse prognoses in CRC patients. **(A-D)** Clinical datasets GSE44861 and GSE44076 depicted mRNA expression levels of TIMP1 and TERT in healthy normal versus CRC tumor groups. **(E-F)** Survival analysis of CRC clinical dataset GSE29623 illustrated the impact of TIMP1 and TERT on patient survival outcomes in CRC.

Supplementary Figure 2 TIMP1 Expression in Normal and CRC (Tumor) Tissues Assessed by H-score Quantification. H-score, calculated as the product of staining intensity (0ten and percentage of positive cells (0llsiveg revealed significantly elevated TIMP1 expression in tumor tissues compared to normal counterparts. ***P*<0.01.

Supplementary Figure 3 The expression of TIMP1 was successfully knocked down by siRNA. HCT-116 and SW480 CRC cells were transiently transfected with TIMP1-specific small interfering RNA (siTIMP1) or negative control siRNA (siControl) using Lipofectamine Transfection Reagent. After 48 hours, whole-cell lysates were prepared and subjected to WB analysis to detect TIMP1 protein levels. β evctin served as an internal control.

Supplementary Figure 4 TIMP1 knockdown significantly elevated both MDA and Fe^2+^ levels. After observing changes in TIMP1 expression levels in CRC cells, differences in Fe^2+^, MDA (ferroptosis indicators) were examined.

Supplementary Figure 5 Knockdown of TIMP1 expression significantly inhibits CRC cell proliferation, whereas the addition of TIMP1 recombinant protein can reverse the low proliferative activity of CRC cells. The CCK8 proliferation assay were utilized to detect the proliferative activity of CRC cells in each group at 24 h, 48 h, and 72 h, respectively.

Supplementary Figure 6 TIMP1 knockdown upregulates ferroptosis-related proteins GPX4 and SLC7A11 while is reversed by DFO treatment. HCT-116 cells were transfected with siRNA targeting TIMP1 (siTIMP1) or negative control siRNA (siControl). Following knockdown, cells were co-treated with the iron chelator deferoxamine (DFO). Protein expression levels of GPX4 and SLC7A11, key regulators of ferroptosis, were analyzed by WB.

Supplementary Figure 7 The stable CRC cell lines with TIMP1 knockdown has been established successfully. HCT-116 and SW480 CRC cells were transduced with lentiviral particles encoding TIMP1-specific short hairpin RNA (TIMP1-KD) or empty control shRNA (Control). Stably transduced cells were selected using tetracycline. Whole-cell lysates were analyzed by Western blot (WB) to assess TIMP1 protein expression.

Supplementary Figure 8 TIMP1 knockdown downregulates ferroptosis suppressors GPX4 and SLC7A11 in CRC xenograft tissues, correlating with enhanced ferroptosis activation. Tumor tissues derived from TIMP1-knockdown (TIMP1-KD) and control xenograft models were lysed and subjected to WB analysis.

## References

[1] Siegel RL Miller KD Wagle NS Jemal A. Cancer statistics, 2023. CA Cancer J Clin. (2023) 73:17–48. doi: 10.3322/caac.21763 36633525

[2] Stockwell BR Jiang X. A physiological function for ferroptosis in tumor suppression by the immune system. Cell Metab. (2019) 30:14–5. doi: 10.1016/j.cmet.2019.06.012 PMC6944065 31269423

[3] Cui W Guo M Liu D Xiao P Yang C Huang H. Gut microbial metabolite facilitates colorectal cancer development via ferroptosis inhibition. Nat Cell Biol. (2024) 6(1):124–37. doi: 10.1038/s41556-023-01314-6 38168770

[4] Zhou TJ Zhang MM Liu DM Huang LL Yu HQ Wang Y. Glutathione depletion and dihydroorotate dehydrogenase inhibition actuated ferroptosis-augment to surmount triple-negative breast cancer. Biomaterials. (2023) 305:122447. doi: 10.1016/j.biomaterials.2023.122447 38154441

[5] Zhang G Mi W Wang C Li J Zhang Y Liu N. Targeting Akt Induced Ferroptosis through Fto/Ythdf2-Dependent Gpx4 M6a Methylation up-Regulating and Degradating in Colorectal Cancer. Cell Death Discov. (2023) 9:457. doi: 10.1038/s41420-023-01746-x 38102129 PMC10724184

[6] Lei G Zhuang L Gan B. The roles of ferroptosis in cancer: tumor suppression, tumor microenvironment, and therapeutic interventions. Cancer Cell. (2024) 42:513–34. doi: 10.1016/j.ccell.2024.03.011 38593779

[7] Li H Sun Y Yao Y Ke S Zhang N Xiong W. Usp8-governed gpx4 homeostasis orchestrates ferroptosis and cancer immunotherapy. Proc Natl Acad Sci U.S.A. (2024) 121:e2315541121. doi: 10.1073/pnas.2315541121 38598341 PMC11032464

[8] Nakamura T Conrad M. Exploiting ferroptosis vulnerabilities in cancer. Nat Cell Biol. (2024) 26(9):1407–19. doi: 10.1038/s41556-024-01425-8 38858502

[9] Justo BL Jasiulionis MG. Characteristics of timp1, cd63, and beta1-integrin and the functional impact of their interaction in cancer. Int J Mol Sci. (2021) 22(17):9319. doi: 10.3390/ijms22179319 34502227 PMC8431149

[10] Xi C Zhang G Sun N Liu M Ju N Shen C. Repurposing homoharringtonine for thyroid cancer treatment through timp1/fak/pi3k/akt signaling pathway. iScience. (2024) 27:109829. doi: 10.1016/j.isci.2024.109829 38770133 PMC11103377

[11] Duch P Diaz-Valdivia N Gabasa M Ikemori R Arshakyan M Fernandez-Nogueira P. Aberrant timp-1 production in tumor-associated fibroblasts drives the selective benefits of nintedanib in lung adenocarcinoma. Cancer Sci. (2024) 115:1505–19. doi: 10.1111/cas.16141 PMC11093210 38476010

[12] Tian Z Tan Y Lin X Su M Pan L Lin L. Arsenic trioxide sensitizes pancreatic cancer cells to gemcitabine through downregulation of the timp1/pi3k/akt/mtor axis. Transl Res. (2023) 255:66–76. doi: 10.1016/j.trsl.2022.11.007 36400307

[13] Tian Z Ou G Su M Li R Pan L Lin X. Timp1 derived from pancreatic cancer cells stimulates schwann cells and promotes the occurrence of perineural invasion. Cancer Lett. (2022) 546:215863. doi: 10.1016/j.canlet.2022.215863 35961511

[14] Hermann CD Schoeps B Eckfeld C Munkhbaatar E Kniep L Prokopchuk O. Timp1 expression underlies sex disparity in liver metastasis and survival in pancreatic cancer. J Exp Med. (2021) 218(11):e20210911. doi: 10.1084/jem.20210911 34533565 PMC8480668

[15] Li X Jiang F Hu Y Lang Z Zhan Y Zhang R. Schisandrin B promotes hepatic stellate cell ferroptosis via wnt pathway-mediated ly6c(Lo) macrophages. J Agric Food Chem. (2023) 71(45):17295–307. doi: 10.1021/acs.jafc.3c03409 37922022

[16] Lai Q Li W Hu D Huang Z Wu M Feng S. Hepatic stellate cell-targeted chemo-gene therapy for liver fibrosis using fluorinated peptide-lipid hybrid nanoparticles. J Control Release. (2024) 376:601–17. doi: 10.1016/j.jconrel.2024.10.044 39437969

[17] Qian R Tang M Ouyang Z Cheng H Xing S. Identification of ferroptosis-related genes in ulcerative colitis: A diagnostic model with machine learning. Ann Transl Med. (2023) 11:177. doi: 10.21037/atm-23-276 36923072 PMC10009563

[18] Gao BB Wang L Li LZ Fei ZQ Wang YY Zou XM. Beneficial effects of oxymatrine from sophora flavescens on alleviating ulcerative colitis by improving inflammation and ferroptosis. J Ethnopharmacol. (2024) 332:118385. doi: 10.1016/j.jep.2024.118385 38797379

[19] Chen C Lan B Xie G Liu Z. Analysis and identification of ferroptosis-related genes in ulcerative colitis. Scand J Gastroenterol. (2023) 58:1422–33. doi: 10.1080/00365521.2023.2240927 37530128

[20] Chen H Sun W Xie M Li Z Chen B Zhang T. Integrated bioinformatics analysis and experimental validation to understand tryptophan metabolism-related genes in hepatocellular carcinoma. J Cancer. (2024) 15(15):4879–92. doi: 10.7150/jca.39132147 PMC11310871 39132147

[21] Zhang J Yan B Spath SS Qun H Cornelius S Guan D. Integrated transcriptional profiling and genomic analyses reveal rpn2 and hmgb1 as promising biomarkers in colorectal cancer. Cell Biosci. (2015) 5:53. doi: 10.1186/s13578-015-0043-9 26388988 PMC4574027

[22] Langfelder P Horvath S. Wgcna: an R package for weighted correlation network analysis. BMC Bioinf. (2008) 9:559. doi: 10.1186/1471-2105-9-559 PMC2631488 19114008

[23] Wilkerson MD Hayes DN. Consensusclusterplus: A class discovery tool with confidence assessments and item tracking. Bioinformatics. (2010) 26:1572–3. doi: 10.1093/bioinformatics/btq170 PMC2881355 20427518

[24] Hanzelmann S Castelo R Guinney J. Gsva: gene set variation analysis for microarray and rna-seq data. BMC Bioinf. (2013) 14:7. doi: 10.1186/1471-2105-14-7 PMC3618321 23323831

[25] Wei R Zhu Y Zhang Y Zhao W Yu X Wang L. Aimp1 promotes multiple myeloma Malignancy through interacting with anp32a to mediate histone H3 acetylation. Cancer Commun (Lond). (2022) 42:1185–206. doi: 10.1002/cac2.12356 PMC9648396 36042007

[26] Wei R Zhong S Qiao L Guo M Shao M Wang S. Steroid 5alpha-reductase type I induces cell viability and migration via nuclear factor-kappab/vascular endothelial growth factor signaling pathway in colorectal cancer. Front Oncol. (2020) 10:1501. doi: 10.3389/fonc.2020.01501 32983992 PMC7484213

[27] Dixon SJ Lemberg KM Lamprecht MR Skouta R Zaitsev EM Gleason CE. Ferroptosis: an iron-dependent form of nonapoptotic cell death. Cell. (2012) 149:1060–72. doi: 10.1016/j.cell.2012.03.042 PMC3367386 22632970

[28] Tang X Hu W You W Fang T. Exploration of key ferroptosis-related genes and immune infiltration in crohn’s disease using bioinformatics. Sci Rep. (2023) 13:12769. doi: 10.1038/s41598-023-40093-w 37550393 PMC10406931

[29] Tan Y Li X Tian Z Chen S Zou J Lian G. Timp1 down-regulation enhances gemcitabine sensitivity and reverses chemoresistance in pancreatic cancer. Biochem Pharmacol. (2021) 189:114085. doi: 10.1016/j.bcp.2020.114085 32522594

[30] Prokopchuk O Grunwald B Nitsche U Jager C Prokopchuk OL Schubert EC. Elevated systemic levels of the matrix metalloproteinase inhibitor timp-1 correlate with clinical markers of cachexia in patients with chronic pancreatitis and pancreatic cancer. BMC Cancer. (2018) 18:128. doi: 10.1186/s12885-018-4055-9 29394913 PMC5797345

[31] Shen Y Ni S Li S Lv B. Role of stemness-related genes timp1, pgf, and snai1 in the prognosis of colorectal cancer through single-cell rna-seq. Cancer Med. (2023) 12:11611–23. doi: 10.1002/cam4.5833 PMC10242850 37017587

[32] Schoeps B Eckfeld C Prokopchuk O Bottcher J Haussler D Steiger K. Timp1 triggers neutrophil extracellular trap formation in pancreatic cancer. Cancer Res. (2021) 81:3568–79. doi: 10.1158/0008-5472.CAN-20-4125 33941611

[33] Meng C Yin X Liu J Tang K Tang H Liao J. Timp-1 is a novel serum biomarker for the diagnosis of colorectal cancer: A meta-analysis. PloS One. (2018) 13:e0207039. doi: 10.1371/journal.pone.0207039 30458003 PMC6245680

[34] Laitinen A Hagstrom J Mustonen H Kokkola A Tervahartiala T Sorsa T. Serum mmp-8 and timp-1 as prognostic biomarkers in gastric cancer. Tumour Biol. (2018) 40:1010428318799266. doi: 10.1177/1010428318799266 30192205

[35] Carpen T Sorsa T Jouhi L Tervahartiala T Haglund C Syrjanen S. High levels of tissue inhibitor of metalloproteinase-1 (Timp-1) in the serum are associated with poor prognosis in hpv-negative squamous cell oropharyngeal cancer. Cancer Immunol Immunother. (2019) 68:1263–72. doi: 10.1007/s00262-019-02362-4 PMC6682571 31240326

[36] Langguth M Maranou E Koskela SA Elenius O Kallionpaa RE Birkman EM. Timp-1 is an activator of mhc-I expression in myeloid dendritic cells with implications for tumor immunogenicity. Genes Immun. (2024) 25:188–200. doi: 10.1038/s41435-024-00274-7 38777826 PMC11178497

[37] Seubert B Grunwald B Kobuch J Cui H Schelter F Schaten S. Tissue inhibitor of metalloproteinases (Timp)-1 creates a premetastatic niche in the liver through sdf-1/cxcr4-dependent neutrophil recruitment in mice. Hepatology. (2015) 61:238–48. doi: 10.1002/hep.27378 PMC4280301 25131778

[38] Meijer MJ Mieremet-Ooms MA van Hogezand RA Lamers CB Hommes DW Verspaget HW. Role of matrix metalloproteinase, tissue inhibitor of metalloproteinase and tumor necrosis factor-alpha single nucleotide gene polymorphisms in inflammatory bowel disease. World J Gastroenterol. (2007) 13:2960–6. doi: 10.3748/wjg.v13.i21.2960 PMC4171149 17589947

