# Supplementary figures and images for: Bioinformatics and experimental unveiling of TIMP1 as a novel therapeutic target in colorectal cancer ferroptosis
Source: Front Oncol. 2025 Jul 4;15:1593107. doi: 10.3389/fonc.2025.1593107 (PMC12270787; doi:10.3389/fonc.2025.1593107)

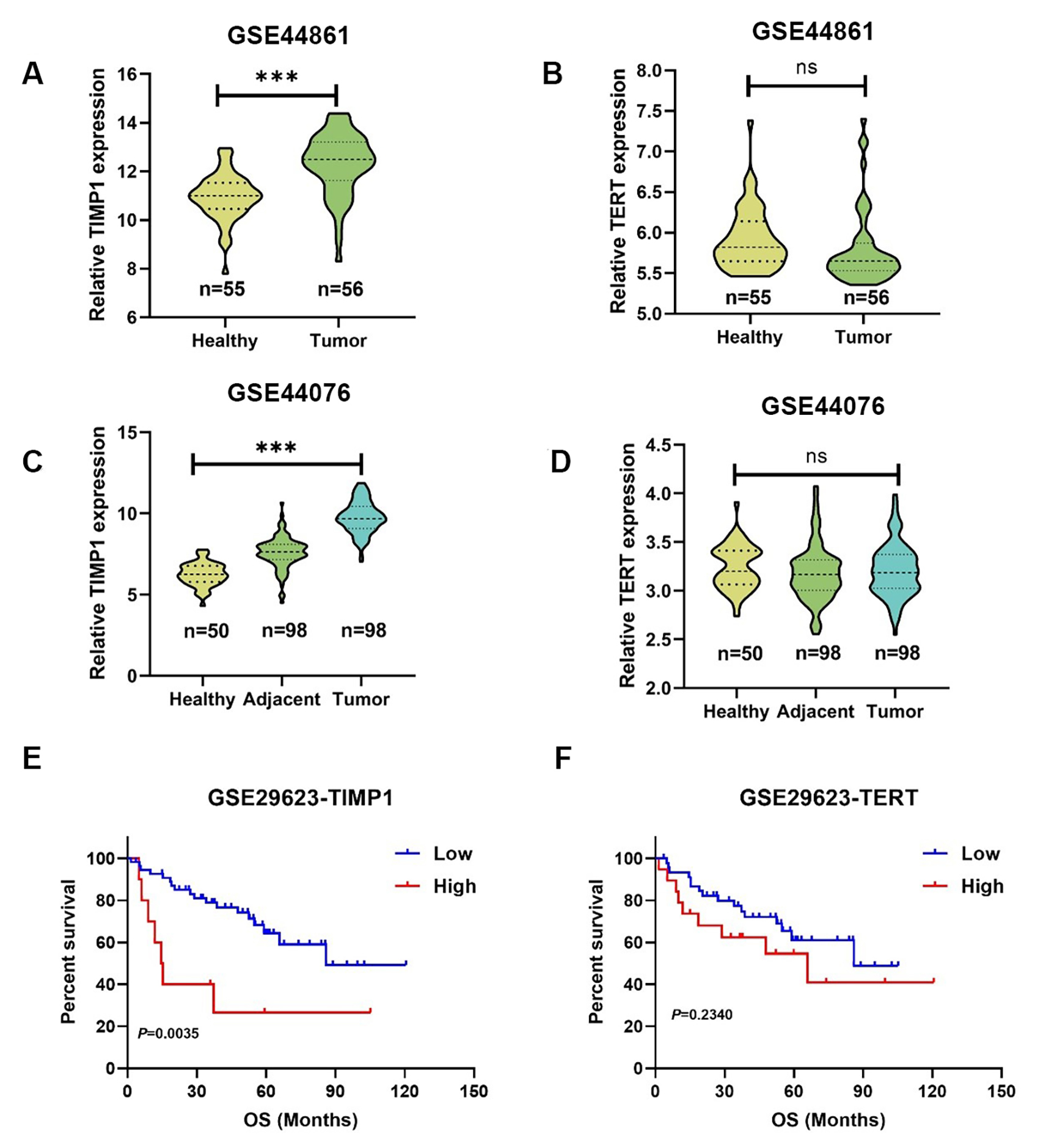

Supplement: Supplementary Figure 1 — Elevated expression of TIMP1, rather than TERT, is closely correlated with adverse prognoses in CRC patients. (A-D) Clinical datasets GSE44861 and GSE44076 depicted mRNA expression levels of TIMP1 and TERT in healthy normal versus CRC tumor groups. (E-F) Survival analysis of CRC clinical dataset GSE29623 illustrated the impact of TIMP1 and TERT on patient survival outcomes in CRC. [file Image1.jpg]

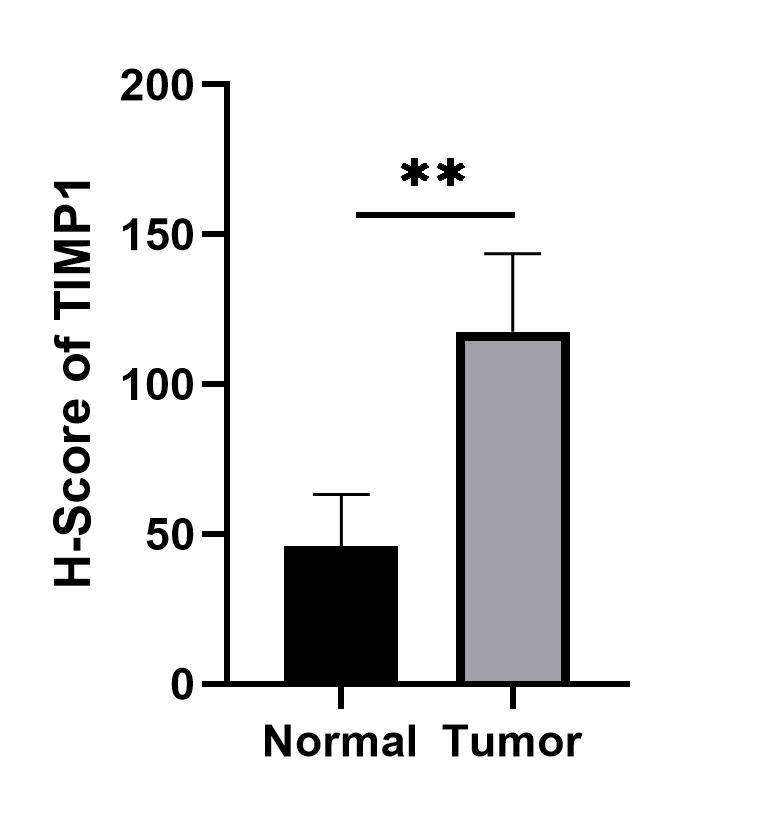

Supplement: Supplementary Figure 2 — TIMP1 Expression in Normal and CRC (Tumor) Tissues Assessed by H-score Quantification. H-score, calculated as the product of staining intensity (0ten and percentage of positive cells (0llsiveg revealed significantly elevated TIMP1 expression in tumor tissues compared to normal counterparts. **P<0.01. [file Image2.jpg]

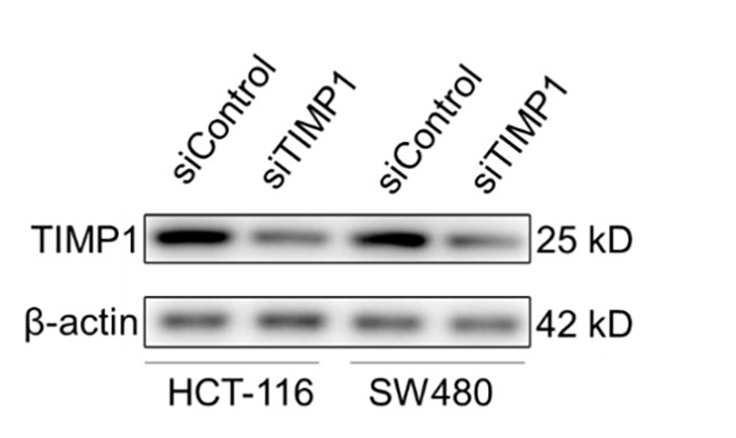

Supplement: Supplementary Figure 3 — The expression of TIMP1 was successfully knocked down by siRNA. HCT-116 and SW480 CRC cells were transiently transfected with TIMP1-specific small interfering RNA (siTIMP1) or negative control siRNA (siControl) using Lipofectamine Transfection Reagent. After 48 hours, whole-cell lysates were prepared and subjected to WB analysis to detect TIMP1 protein levels. β evctin served as an internal control. [file Image3.jpg]

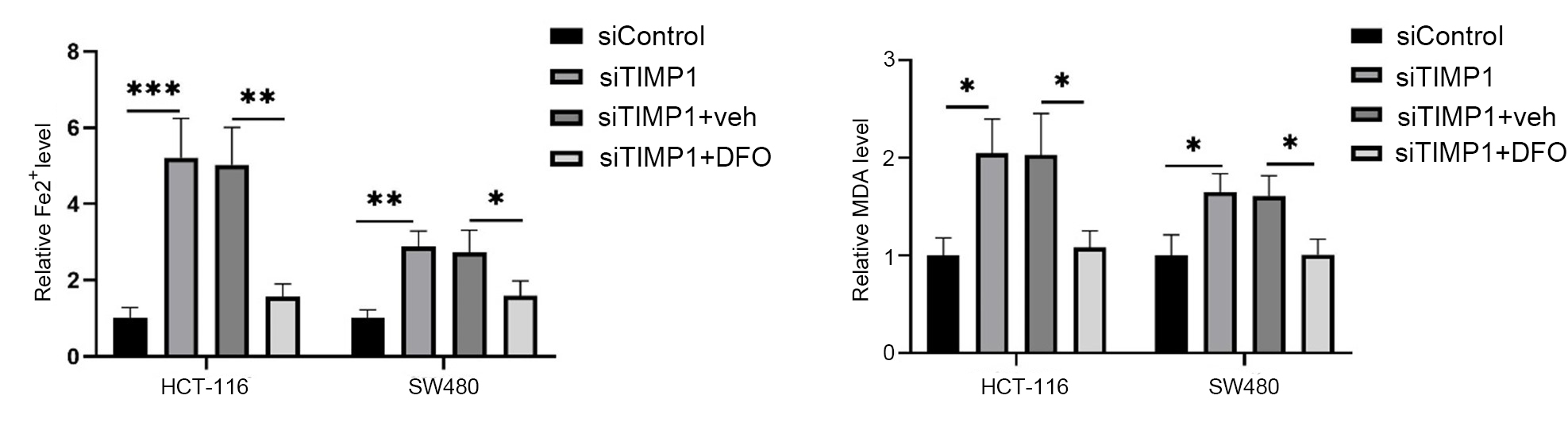

Supplement: Supplementary Figure 4 — TIMP1 knockdown significantly elevated both MDA and Fe2+ levels. After observing changes in TIMP1 expression levels in CRC cells, differences in Fe2+, MDA (ferroptosis indicators) were examined. [file Image4.jpg]

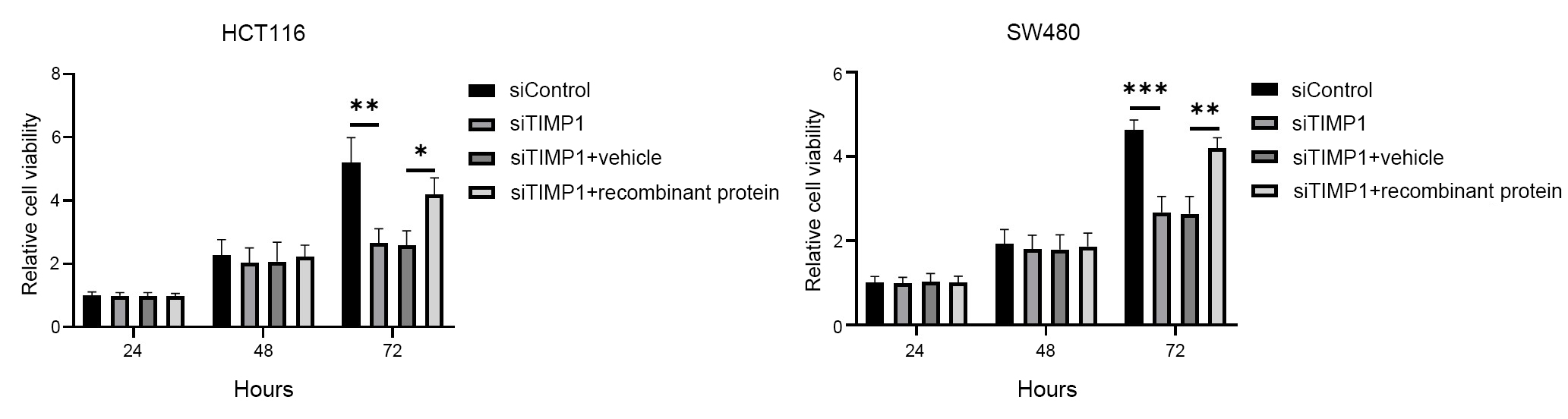

Supplement: Supplementary Figure 5 — Knockdown of TIMP1 expression significantly inhibits CRC cell proliferation, whereas the addition of TIMP1 recombinant protein can reverse the low proliferative activity of CRC cells. The CCK8 proliferation assay were utilized to detect the proliferative activity of CRC cells in each group at 24 h, 48 h, and 72 h, respectively. [file Image5.jpg]

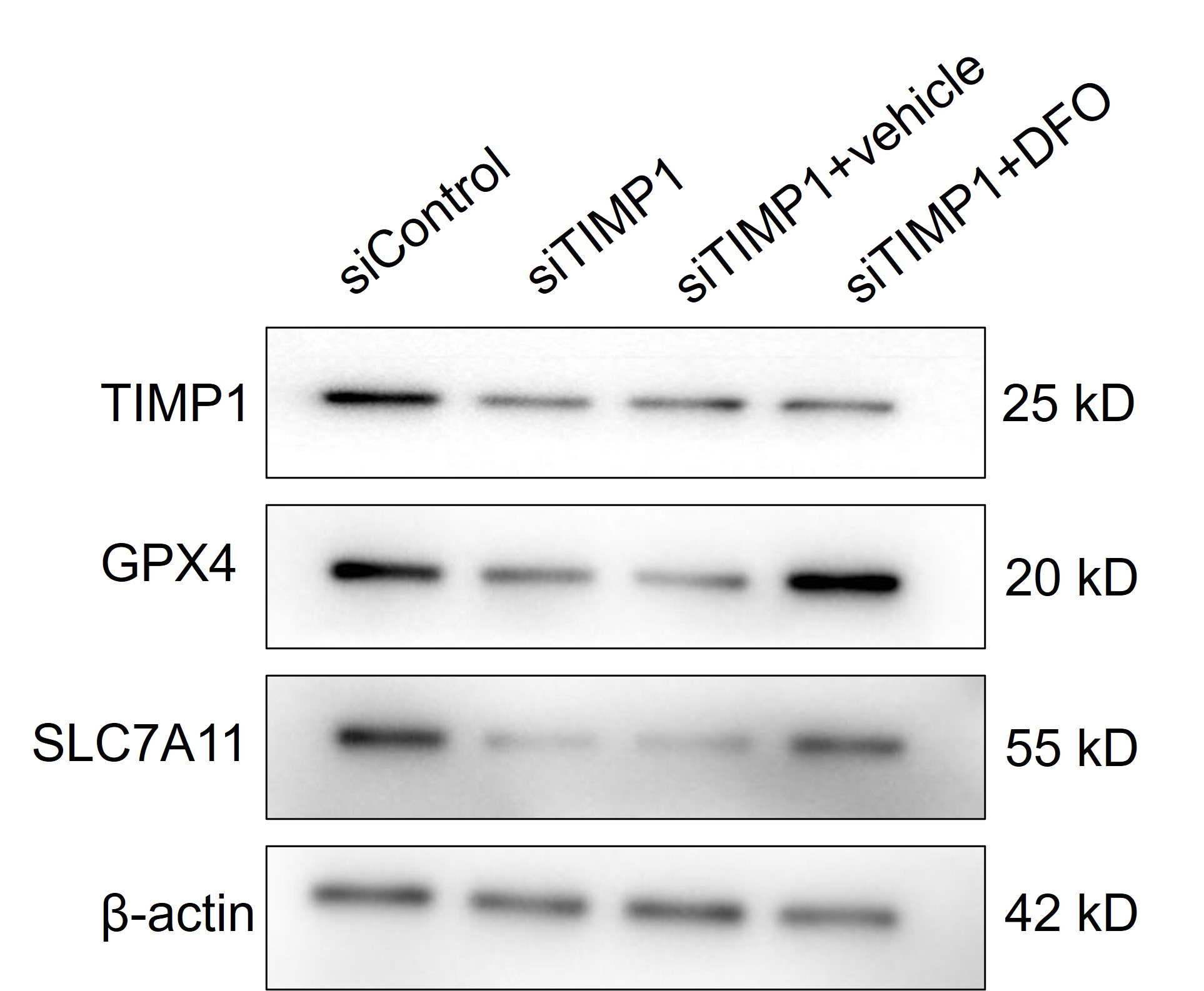

Supplement: Supplementary Figure 6 — TIMP1 knockdown upregulates ferroptosis-related proteins GPX4 and SLC7A11 while is reversed by DFO treatment. HCT-116 cells were transfected with siRNA targeting TIMP1 (siTIMP1) or negative control siRNA (siControl). Following knockdown, cells were co-treated with the iron chelator deferoxamine (DFO). Protein expression levels of GPX4 and SLC7A11, key regulators of ferroptosis, were analyzed by WB. [file Image6.jpg]

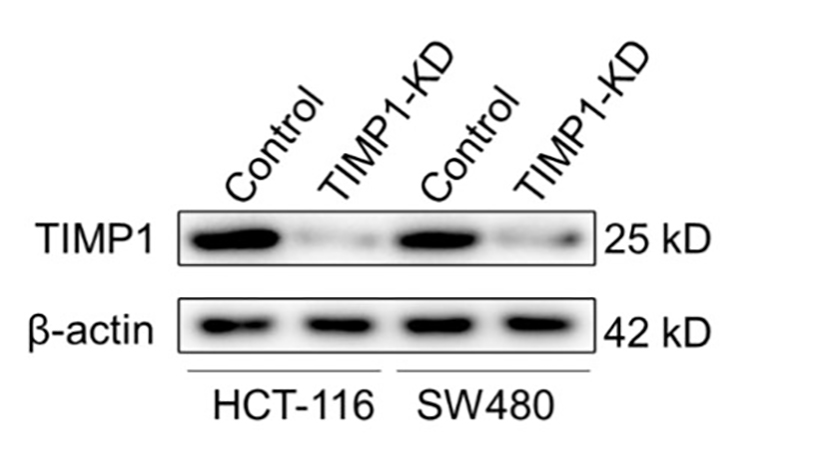

Supplement: Supplementary Figure 7 — The stable CRC cell lines with TIMP1 knockdown has been established successfully. HCT-116 and SW480 CRC cells were transduced with lentiviral particles encoding TIMP1-specific short hairpin RNA (TIMP1-KD) or empty control shRNA (Control). Stably transduced cells were selected using tetracycline. Whole-cell lysates were analyzed by Western blot (WB) to assess TIMP1 protein expression. [file Image7.jpg]

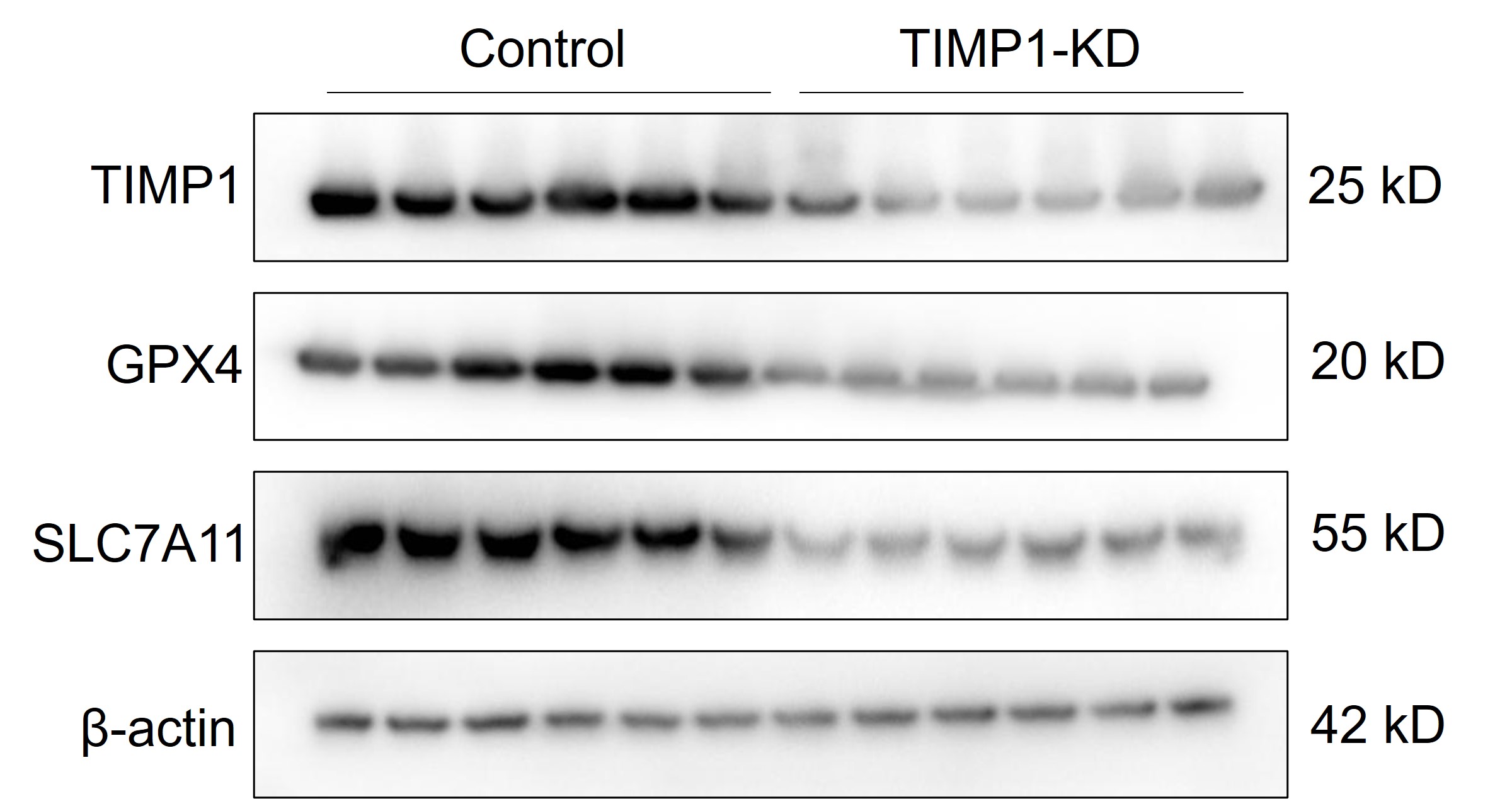

Supplement: Supplementary Figure 8 — TIMP1 knockdown downregulates ferroptosis suppressors GPX4 and SLC7A11 in CRC xenograft tissues, correlating with enhanced ferroptosis activation. Tumor tissues derived from TIMP1-knockdown (TIMP1-KD) and control xenograft models were lysed and subjected to WB analysis. [file Image8.jpg]
