# Supplementary material for: Bioinformatics and experimental unveiling of TIMP1 as a novel therapeutic target in colorectal cancer ferroptosis
Source: Front Oncol. 2025 Jul 4;15:1593107. doi: 10.3389/fonc.2025.1593107 (PMC12270787; doi:10.3389/fonc.2025.1593107)
Supplement: Supplementary file 9 [file DataSheet1.docx]

**Supplementary Figure S1: Elevated expression of TIMP1, rather than TERT, is closely correlated with adverse prognoses in CRC patients.** (A-D) Clinical datasets GSE44861 and GSE44076 depicted mRNA expression levels of TIMP1 and TERT in healthy normal versus CRC tumor groups. (E-F) Survival analysis of CRC clinical dataset GSE29623 illustrated the impact of TIMP1 and TERT on patient survival outcomes in CRC.

**Supplementary Figure S2: TIMP1 Expression in Normal and CRC (Tumor) Tissues Assessed by H-score Quantification.** H-score, calculated as the product of staining intensity (0–3) and percentage of positive cells (0–100%), revealed significantly elevated TIMP1 expression in tumor tissues compared to normal counterparts.

**Supplementary Figure S3: The expression of TIMP1 was successfully knocked down by siRNA.** HCT-116 and SW480 CRC cells were transiently transfected with TIMP1-specific small interfering RNA (siTIMP1) or negative control siRNA (siControl) using Lipofectamine Transfection Reagent. After 48 hours, whole-cell lysates were prepared and subjected to WB analysis to detect TIMP1 protein levels. β-actin served as an internal control.

**Supplementary Figure S4: TIMP1 knockdown significantly elevated both MDA and Fe²⁺ levels.** After observing changes in TIMP1 expression levels in CRC cells, differences in Fe^2+^, MDA (ferroptosis indicators) were examined.

**Supplementary Figure S5: Knockdown of TIMP1 expression significantly inhibits CRC cell proliferation, whereas the addition of TIMP1 recombinant protein can reverse the low proliferative activity of CRC cells.** The CCK8 proliferation assay were utilized to detect the proliferative activity of CRC cells in each group at 24 h, 48 h, and 72 h, respectively.

**Supplementary Figure S6: TIMP1 knockdown upregulates ferroptosis-related proteins GPX4 and SLC7A11 while is reversed by DFO treatment.** HCT-116 cells were transfected with siRNA targeting TIMP1 (siTIMP1) or negative control siRNA (siControl). Following knockdown, cells were co-treated with the iron chelator deferoxamine (DFO). Protein expression levels of GPX4 and SLC7A11, key regulators of ferroptosis, were analyzed by Western blot.

**Supplementary Figure S7:** **The stable CRC cell lines with TIMP1 knockdown has been established successfully.** HCT-116 and SW480 CRC cells were transduced with lentiviral particles encoding TIMP1-specific short hairpin RNA (TIMP1-KD) or empty control shRNA (Control). Stably transduced cells were selected using tetracycline. Whole-cell lysates were analyzed by Western blot (WB) to assess TIMP1 protein expression.

**Supplementary Figure S8: TIMP1 knockdown downregulates ferroptosis suppressors GPX4 and SLC7A11 in CRC xenograft tissues, correlating with enhanced ferroptosis activation.** Tumor tissues derived from TIMP1-knockdown (TIMP1-KD) and control xenograft models were lysed and subjected to WB analysis.
